# Supplementary material for: Time-surrogate variables enhance the association between cardiotocographic features and intrapartum hypoxic-ischemic encephalopathy
Source: PLOS Digit Health. 2026 Aug 21;5(8):e0001111. doi: 10.1371/journal.pdig.0001111 (PMC13497259; doi:10.1371/journal.pdig.0001111)
Supplement: Supplementary file 1 [file pdig.0001111.s007.docx]

### **Response to Reviewers**

**Dear Editor and Reviewers,**

We would like to thank the Academic Editor and the Reviewers for their comprehensive evaluation of our manuscript, "Time-Surrogate Variables Enhance the Association Between Cardiotocographic Features and Intrapartum Hypoxic-Ischemic Encephalopathy" (PDIG-D-25-01012). The provided comments and critiques were insightful and have helped to substantially strengthen our work.

Below, we provide a point-by-point response addressing each of the concerns that were raised. Changes made to the manuscript have been highlighted in the revised tracked-changes document.

### **Responses to the Academic Editor**

**Editor Comment:** *This manuscript is interesting, scientifically sound, and very well-written. However, a main methodological decision (exclusion of CS cases) has been raised by both Reviewers and I personally agree with them that this choice is somehow questionable. I think that including those cases too is relevant, either with all the others or as a separate group, or both. In any case, I strongly suggest the authors to provide a clear rebuttal letter to the Reviewers' comments, along with the revised version.*

**Author Response:** We thank the Editor for his constructive comments. We agree that excluding Caesarean section (CS) cases limits the clinical scope of the paper, given that CS is the primary intervention for suspected fetal compromise. Following the suggestions from the Editor and the Reviewers, we have now fully analyzed the CS cohort as a separate group, stratifying them by the same outcome criteria. We opted for a separate cohort design rather than pooling because the clinical meaning of the time of birth is different between vaginal and Caesarean births, as detailed in the Discussion. This new analysis (detailed in our responses below) confirms that Time from Labor Onset (TLO) remains the strongest and most robust time-surrogate variable, even when the natural culmination of labor is altered by surgical intervention.

**Action:** The conditional NMI analysis was run separately on the 21,425 Caesarean cases. A small description of the separate analysis was included to the Methods section and the resulting NMI plot was added to the Results section.

### **Responses to Reviewer #1**

**Reviewer 1, Point 1:** *...Actually, this decision [to exclude Caesarean deliveries] may have removed the essential data from the study. In case you would like to make the claim that the CTG can be used to signal fetal distress that necessitates a Caesarean delivery, your study population should include cases with Caesarean deliveries. Also the question arises: why did the deliveries of the 1793 acidosis and 155 HIE newborns that you included in the study not meet the criteria for a Caesarean delivery? These issues should be addressed in the paper.*

**Author Response:** We deeply appreciate the Reviewer highlighting this limitation in our initial submission. We agree that excluding CS cases could bias the results, highlighting TSVs that are only advantageous for vaginal deliveries.

Our initial rationale for isolating vaginal deliveries was to compare TSVs up to the natural conclusion of labor, avoiding alignment issues that stem from having a time of delivery that results from clinical decisions. However, to address your point, we have now performed a complete parallel analysis of the 21,425 Caesarean delivery cases. Treating them as a separate cohort, we evaluated the performance of the proposed time-surrogate variables (TSVs). The results, now included in the Results Section (Fig 6) and Figures S1-S3, demonstrate that time from labor onset (TLO) remains the most informative and robust TSV across all outcome groups, regardless of delivery method.

Regarding your question as to why the 1,793 acidosis and 155 HIE cases did not end in a CS, clinical practice is complex, and pathological outcomes in vaginal deliveries typically occur for multiple reasons. For instance, severe fetal compromise can develop rapidly during the final phase of the second stage of labor, at which point an operative vaginal delivery is faster than moving to an operating room for a CS. Moreover, in these cases a CS may be nearly impossible if the head of the fetus has started descending the birth canal. Second, unfortunately, these outcomes sometimes reflect a failure of the clinical team to recognize non-reassuring CTG patterns early enough to intervene. This underscores the exact clinical need for our proposed automated time-aware analysis. We have added a paragraph detailing this clinical context to the Discussion.

**Action:** The conditional NMI analysis was run separately on the 21,425 Caesarean cases. A small description of the separate analysis was included to the Methods section and the resulting NMI plot was added to the Results section. Also, we have expanded the Discussion to explain why pathological cases sometimes end in vaginal delivery (such as rapid deterioration late in the second stage, or failure of clinical staff to recognize CTG patterns in time).

**Reviewer 1, Point 2:** *Line 379: I have some difficulties with the phrase: "Birth weight distributions were similar across groups (p=0.0739)." I think the distributions were not statistically significantly different, what is not the same as similar.*

**Author Response:** We thank the Reviewer for pointing out this imprecision in our statistical terminology. We would like to inform you that after including Caesarean deliveries in our analysis, now birth weight is also different across groups. We have updated our Results section to reflect this new data.

**Action:** Birth weight description was updated.

**Reviewer 1, Point 3:** *Finally, the abstract would benefit from the addition of some quantitative data describing the major findings and supporting the conclusions.*

**Author Response:** We agree with this suggestion. We have revised the abstract to highlight the increase in NMI for the top ten most informative baseline features, which was over 20%.

**Action:** Added comparison of TSV vs no TSV to the abstract

### **Responses to Reviewer #2**

**Reviewer 2, Point 1:** *Major: The authors exclude CS births... the dataset becomes biased against prolonged labour... and also against identifiably abnormal CTG traces. I would strongly recommend adding CS births to the analysis, and including CS healthy / acidosis / compromised as separate groups, akin to the blood gases stratification.*

**Author Response:** We thank the Reviewer for their valuable suggestion. We agree that our initial exclusion could introduce bias. Following your recommendation, we have incorporated the CS births into the analysis as a distinct, separate cohort stratified by the exact same outcome groups. As detailed in our response to Reviewer 1 and in the Results section of the revised manuscript, this addition validated that TLO is robust and applicable even when natural labor progression is interrupted by surgical intervention.

**Action:** See actions detailed under Response to Reviewer 1 and Response to Editor, Point 1.

**Reviewer 2, Point 2:** *Another major concern I have is around the definition of labour onset. How was labour onset defined? ... As TLO is central to this paper, an explicit definition should be included in Methods.*

**Author Response:** This is a very important point. In our revised manuscript, we have explicitly detailed the definition of labor onset in the Methods section under the “Definition of labor onset” subsection. Because our dataset relies on retrospective electronic health records, labor onset was defined algorithmically using a combination of uterine pressure (UP) signals and documented clinical examinations.

Specifically, the algorithm evaluated 20-minute epochs for the presence of contractions. If contractions were present, labor onset was triggered by either the rupture of membranes, cervical dilation exceeding 4 cm, or any documented change in cervical effacement or dilation. If UA data was missing, the onset was determined based solely on the cervical exam milestones.

**Action:** Method section was expanded with a new “Definition of labor onset” subsection.

**Reviewer 2, Point 3:** *Relatedly, how often was onset spontaneous as opposed to induced and have you done any analysis stratified by labour onset method?*

**Author Response:** We thank the Reviewer for raising this important clinical point. We have updated Table 2 to report the rates of labor induction across all outcome groups and both delivery cohorts. Labor induction was identified retrospectively via electronic health records by the inpatient administration of labor-inducing pharmacological agents (e.g., oxytocin, misoprostol, dinoprostone) prior to the identified onset of labor.

While the chi-square test reached significance owing to the large sample size, the absolute differences in induction rates across outcome groups were modest (ranging from 16.4% to 22.88%).

TLO measures cumulative exposure regardless of how contractions were initiated, and thus formal stratification by labor onset method is not required for the primary analysis. Moreover, the small number of induced HIE cases presents a statistical barrier to this approach in our database: the HIE group contains only 65 induced cases, of which 35 were delivered vaginally and 30 via Caesarean sections. Our bootstrapping procedure is stratified by 15 hospitals to preserve intra-hospital variability. Distributing 35 induced vaginal HIE cases (or 30 Caesarean) across 15 hospitals would leave several hospitals with zero or one case per stratum, making stable NMI estimation infeasible even if we increase the number of bootstrapping samples (empty subgroups). However, we note that the similarity in induction rates across groups provides indirect evidence that induction status is unlikely to confound our primary findings. Thus, suggesting that our findings are robust to the labor onset method, even though formal stratification was not statistically possible.

**Action:** Included the rate of labor induction per group in Table 2.

**Reviewer 2, Point 4:** *Minor: Were instrumental births (forceps, ventouse) included in the analysis? This relates to Q1 above.*

**Author Response:** Yes, instrumental vaginal deliveries (forceps and vacuum extraction) were included within the "vaginal delivery" cohort. We have clarified this inclusion criteria explicitly in the Study Population subsection of the Methods and include the rate of operative deliveries in Table 2.

**Action:** Expanded inclusion criteria to mention inclusion of forceps and vacuum extraction and updated Table 2.

**Reviewer 2, Point 5:** *Why were late term births not included? I understand excluding postterm births of >=42 weeks gestation, but births at 41 weeks gestation account for about 11% of all births here in the UK, the majority of which I believe are clinically managed like term births.*

**Author Response:** We thank the Reviewer for catching this discrepancy. The stated upper limit of 40 weeks in the manuscript text was a typo that carried over from earlier in the project.

In reality, our dataset includes late-term births infants that went into labor with a gestational age greater than 35 weeks and lower than 44 weeks (maximum found was 43 weeks 5 days), which is consistent with the comprehensive methodology detailed in our dataset description (Kearney et al., *MethodsX*, 2024).

To clarify, 36.5% (63,596/174,186) of the infants in our analyzed cohort had a gestational age strictly greater than 40 weeks. Specifically, 0.24% (419/174,186) had a gestational age between 42 and 44 weeks. These 419 late-term cases were distributed across our outcome groups as follows: 1 HIE, 14 Acidosis, 152 Healthy with blood gas, and 252 Healthy without blood gas.

We apologize for any confusion this typo caused and have corrected the inclusion criteria in the Methods section to accurately state the 35–44 week gestational age range, ensuring the text now accurately reflects the data analyzed.

**Action:** The gestational age range in the Study Population subsection was updated to 35 - 44 weeks.

**Reviewer 2, Point 6:** *In results, the authors report the relationship between TSVs and CTG features. It would be interesting to see the relationship between different TSVs, stratified by outcome group (e.g. via 2-d density plots as SI). I would be curious to see TLO vs CCT in particular, as this may be an interesting measure of labour progress.*

**Author Response:** We thank the Reviewer for this excellent idea. Visualizing the interaction between TSVs provides great insight into labor dynamics. We have generated 2D conditional density plots comparing CCT against TLO, stratified by the four outcome groups. These plots illustrate the probability density of the CCT variable as TLO increases. The plots are available in the Supplementary Information (Figure S4 and S5). As it is expected, they show a positive correlation between CCT and TLO, which is maintained across all outcome groups.

**Action:** New 2D conditional density plots show p(CCT|TLO) in Supporting Information document (Fig S4 and S5).

**Reviewer 2, Point 7:** *Please ensure figures are colourblind-friendly (in particular red/green).*

**Author Response:** Thank you for pointing this out. We have completely redesigned the color palettes for all figures that had red and green colors to utilize a colorblind-safe palette, ensuring accessibility for all readers.

**Action:** Updated color palettes in all figures.
