# Supplementary material for: Time-surrogate variables enhance the association between cardiotocographic features and intrapartum hypoxic-ischemic encephalopathy
Source: PLOS Digit Health. 2026 Aug 21;5(8):e0001111. doi: 10.1371/journal.pdig.0001111 (PMC13497259; doi:10.1371/journal.pdig.0001111)
Supplement: Peer review history

# Peer review: Decision Letter 0

**DOI:** 10.1371/journal.pdig.0001111.r001

**Authors:** Leo Anthony Celi, Danilo Pani

19 Mar 2026

PDIG-D-25-01012

Time-Surrogate Variables Enhance the Association Between Cardiotocographic Features and Intrapartum Hypoxic-Ischemic Encephalopathy

PLOS Digital Health

Dear Dr. Vargas-Calixto,

Thank you for submitting your manuscript to PLOS Digital Health. After careful consideration, we feel that it has merit but does not fully meet PLOS Digital Health's publication criteria as it currently stands. Therefore, we invite you to submit a revised version of the manuscript that addresses the points raised during the review process.

Please submit your revised manuscript by May 18 2026 11:59PM. If you will need more time than this to complete your revisions, please reply to this message or contact the journal office at digitalhealth@plos.org. When you're ready to submit your revision, log on to https://www.editorialmanager.com/pdig/ and select the 'Submissions Needing Revision' folder to locate your manuscript file.

Please include the following items when submitting your revised manuscript:

* A letter that responds to each point raised by the editor and reviewer(s). You should upload this letter as a separate file labeled 'Response to Reviewers'. This file does not need to include responses to any formatting updates and technical items listed in the 'Journal Requirements' section below.

* A marked-up copy of your manuscript that highlights changes made to the original version. You should upload this as a separate file labeled 'Revised Manuscript with Track Changes'.

* An unmarked version of your revised paper without tracked changes. You should upload this as a separate file labeled 'Manuscript'.

If you would like to make changes to your financial disclosure, competing interests statement, or data availability statement, please make these updates within the submission form at the time of resubmission. Guidelines for resubmitting your figure files are available below the reviewer comments at the end of this letter.

We look forward to receiving your revised manuscript.

Kind regards,

Danilo Pani, Ph.D.

Academic Editor

PLOS Digital Health

Danilo Pani

Academic Editor

PLOS Digital Health

Leo Anthony Celi

Editor-in-Chief

PLOS Digital Health

orcid.org/0000-0001-6712-6626

**Journal Requirements:**

1. We ask that a manuscript source file is provided at Revision. Please upload your manuscript file as a .doc, .docx, .rtf or .tex.

2. Please provide separate figure files in .tif or .eps format.

For more information about figure files please see our guidelines:  LINK

https://journals.plos.org/digitalhealth/s/figures

https://journals.plos.org/digitalhealth/s/figures#loc-file-requirements

If the reviewer comments include a recommendation to cite specific previously published works, please review and evaluate these publications to determine whether they are relevant and should be cited. There is no requirement to cite these works unless the editor has indicated otherwise.

**Additional Editor Comments (if provided):**

This manuscript is interesting, scientifically sound, and very well-written. However, a main methodological decision (exclusion of CS cases) has been raised by both Reviewers and I personally agree with them that this choice is somehow questionable. I think that including those cases too is relevant, either with all the others or as a separate group, or both. In any case, I strongly suggest the authors to provide a clear rebuttal letter to the Reviewers' comments, along with the revised version.

[Note: HTML markup is below. Please do not edit.]

**Reviewers' Comments:**

Reviewer's Responses to Questions

**Comments to the Author**

1. Does this manuscript meet PLOS Digital Health’s publication criteria? Is the manuscript technically sound, and do the data support the conclusions? The manuscript must describe methodologically and ethically rigorous research with conclusions that are appropriately drawn based on the data presented.

Reviewer #1: Yes

Reviewer #2: Yes

**********

2. Has the statistical analysis been performed appropriately and rigorously?

Reviewer #1: Yes

Reviewer #2: Yes

**********

3. Have the authors made all data underlying the findings in their manuscript fully available (please refer to the Data Availability Statement at the start of the manuscript PDF file)?

The PLOS Data policy requires authors to make all data underlying the findings described in their manuscript fully available without restriction, with rare exception. The data should be provided as part of the manuscript or its supporting information, or deposited to a public repository. For example, in addition to summary statistics, the data points behind means, medians and variance measures should be available. If there are restrictions on publicly sharing data—e.g. participant privacy or use of data from a third party—those must be specified.

Reviewer #1: No

Reviewer #2: Yes

**********

4. Is the manuscript presented in an intelligible fashion and written in standard English?

PLOS Digital Health does not copyedit accepted manuscripts, so the language in submitted articles must be clear, correct, and unambiguous. Any typographical or grammatical errors should be corrected at revision, so please note any specific errors here.

Reviewer #1: Yes

Reviewer #2: Yes

**********

5. Review Comments to the Author

Please use the space provided to explain your answers to the questions above. You may also include additional comments for the author, including concerns about dual publication, research ethics, or publication ethics. (Please upload your review as an attachment if it exceeds 20,000 characters)

Reviewer #1: With interest I studied the paper “Time-Surrogate Variables Enhance the Association Between Cardiotocographic Features and Intrapartum Hypoxic-Ischemic Encephalopathy” by Johann Vargas-Calixto et al., who studied CTG data and delivery outcomes in 152,761 pregnant woman. This is really an impressive group of deliveries, allowing for detailed analysis and conclusions. They found that the predictability of complications on the basis of the CTG was specifically better when the time since labor onset was included in the analysis, a conclusion that seems logical.

In my view, the paper is clearly written, and the methodology is sound except for the decision to exclude Caesarean deliveries from the study population. As the authors state on lines 95-96: “Additionally, we excluded fetuses delivered via Caesarean section to focus exclusively on the natural progression and culmination of labor for TSV comparisons”, though on the other hand (lines 30-31) “The goal of CTG is to assess fetal tolerance to labor by identifying FHR patterns that indicate significant fetal hypoxemia, so that timely interventions, such as emergency Caesarean deliveries, can prevent progression to organ injury and neonatal HIE.” Actually, this decision may have removed the essential data from the study. In case you would like to make the claim that the CTG can be used to signal fetal distress that necessitates a Caesarean delivery, your study population should include cases with Caesarean deliveries. Also the question arises: why did the deliveries of the 1793 acidosis and 155 HIE newborns that you included in the study not meet the criteria for a Caesarean delivery? These issues should be addressed in the paper.

Another way to look at this issue would be to applying your analysis to the CTGs made in cases with Caesarean deliveries.

Line 379: I have some difficulties with the phrase: “Birth weight distributions were similar across groups (p = 0.0736).” I think the distributions were not statistically significantly different, what is not the same as similar.

Finally, the abstract would benefit from the addition of some quatitative data describing the major findings and supporting the conclusions.

Reviewer #2: In their manuscript, Vargas—Calixto et al. draw attention to an underdiscussed issue in computerised CTG analysis. We, often implicitly, tend to treat the fetal heart rate trace as time-invariant, while labour itself is obviously not. It therefore stands to reason that some measure of labour progress (e.g. time from labour onset) may improve CTG analysis and our ability to identify fetuses at risk of hypoxia. This is of great clinical importance, as timely and appropriate intervention in labour can prevent HIE.

Overall, I found the manuscript clear, well-argued and comprehensive. The scale of data used is commendable. However, I do have a major concern around inclusion/exclusion criteria, as well as some other more minor questions.

Major:

1. The authors exclude CS births in order to “focus exclusively on the natural progression and culmination of labor” (L95—96), which is understandable rationale. However, by excluding CS cases, the authors have biased their dataset significantly, in two ways. The two most common primary reasons listed for emergency CS are prolonged labour, and presumed fetal compromise. By excluding CS cases (ca.20% of all births in their data, Fig. 1), the dataset becomes biased against prolonged labour (by definition time-dependent and not known at labour onset), and also against identifiably abnormal CTG traces (as abnormal / worrying CTG are more likely to lead to an emergency CS).

There is a similar bias in the data that the authors explore explicitly, with blood gases. Like an emergency CS, the measurement of blood gases occurs when there is suspicion of possible fetal compromise. In Figure 3, the authors illustrate a clear difference in feature entropy in the deceleration features among healthy births with (green) and without (black) blood gases taken. One can imagine a similar trend may be observed between the CS and vaginal births.

I would strongly recommend adding CS births to the analysis, and including CS healthy / acidosis / compromised as separate groups, akin to the blood gases stratification. As it stands, the dataset excluding CS is difficult to interpret clinically.

2. Another major concern I have is around the definition of labour onset. How was labour onset defined? Many women spend early labour at home and onset of labour can be hard to define unambiguously. As TLO is central to this paper, an explicit definition should be included in Methods. Relatedly, how often was onset spontaneous as opposed to induced and have you done any analysis stratified by labour onset method?

Minor:

3. Were instrumental births (forceps, ventouse) included in the analysis? This relates to Q1 above.

4. Why were late term births not included? I understand excluding postterm births of >=42 weeks gestation, but births at 41 weeks gestation account for about 11% of all births here in the UK, the majority of which I believe are clinically managed like term births.

5. In results, the authors report the relationship between TSVs and CTG features. It would be interesting to see the relationship between different TSVs, stratified by outcome group (e.g. via 2-d density plots as SI). I would be curious to see TLO vs CCT in particular, as this may be an interesting measure of labour progress.

6. Please ensure figures are colourblind-friendly (in particular red/green).

**********

6. PLOS authors have the option to publish the peer review history of their article (what does this mean?). If published, this will include your full peer review and any attached files.

**Do you want your identity to be public for this peer review?**  If you choose “no”, your identity will remain anonymous but your review may still be made public.

For information about this choice, including consent withdrawal, please see our Privacy Policy.

Reviewer #1: No

Reviewer #2: No

**********

[NOTE: If reviewer comments were submitted as an attachment file, they will be attached to this email and accessible via the submission site. Please log into your account, locate the manuscript record, and check for the action link "View Attachments". If this link does not appear, there are no attachment files.]

**Figure resubmission:**

While revising your submission, we strongly recommend that you use PLOS’s NAAS tool (https://ngplosjournals.pagemajik.ai/artanalysis) to test your figure files. NAAS can convert your figure files to the TIFF file type and meet basic requirements (such as print size, resolution), or provide you with a report on issues that do not meet our requirements and that NAAS cannot fix.

After uploading your figures to PLOS’s NAAS tool - https://ngplosjournals.pagemajik.ai/artanalysis, NAAS will process the files provided and display the results in the "Uploaded Files" section of the page as the processing is complete. If the uploaded figures meet our requirements (or NAAS is able to fix the files to meet our requirements), the figure will be marked as "fixed" above. If NAAS is unable to fix the files, a red "failed" label will appear above. When NAAS has confirmed that the figure files meet our requirements, please download the file via the download option, and include these NAAS processed figure files when submitting your revised manuscript.

**Reproducibility:**

To enhance the reproducibility of your results, we recommend that authors of applicable studies deposit laboratory protocols in protocols.io, where a protocol can be assigned its own identifier (DOI) such that it can be cited independently in the future. Additionally, PLOS ONE offers an option to publish peer-reviewed clinical study protocols. Read more information on sharing protocols at https://plos.org/protocols?utm_medium=editorial-email&utm_source=authorletters&utm_campaign=protocols

# Author comment: Author response to Decision Letter 1

**DOI:** 10.1371/journal.pdig.0001111.r002

2 Apr 2026

Attachment Submitted filename: response_to_reviewers.docx

# Peer review: Decision Letter 1

**DOI:** 10.1371/journal.pdig.0001111.r003

**Authors:** Leo Anthony Celi, Danilo Pani

19 Jun 2026

Time-Surrogate Variables Enhance the Association Between Cardiotocographic Features and Intrapartum Hypoxic-Ischemic Encephalopathy

PDIG-D-25-01012R1

Dear Ph.D. Vargas-Calixto,

We are pleased to inform you that your manuscript 'Time-Surrogate Variables Enhance the Association Between Cardiotocographic Features and Intrapartum Hypoxic-Ischemic Encephalopathy' has been provisionally accepted for publication in PLOS Digital Health.

Before your manuscript can be formally accepted you will need to complete some formatting changes, which you will receive in a follow-up email from a member of our team.

Please note that your manuscript will not be scheduled for publication until you have made the required changes, so a swift response is appreciated.

IMPORTANT: The editorial review process is now complete. PLOS will only permit corrections to spelling, formatting or significant scientific errors from this point onwards. Requests for major changes, or any which affect the scientific understanding of your work, will cause delays to the publication date of your manuscript.

If your institution or institutions have a press office, please notify them about your upcoming paper to help maximize its impact. If they'll be preparing press materials, please inform our press team as soon as possible -- no later than 48 hours after receiving the formal acceptance. Your manuscript will remain under strict press embargo until 2 pm Eastern Time on the date of publication. For more information, please contact digitalhealth@plos.org.

Thank you again for supporting Open Access publishing; we are looking forward to publishing your work in PLOS Digital Health.

Best regards,

Danilo Pani, Ph.D.

Academic Editor

PLOS Digital Health

***********************************************************

**Additional Editor Comments (if provided):**

All the comments have been addressed and the manuscript can be considered for publication.

**Reviewer Comments (if any, and for reference):**

Reviewer's Responses to Questions

**Comments to the Author**

1. If the authors have adequately addressed your comments raised in a previous round of review and you feel that this manuscript is now acceptable for publication, you may indicate that here to bypass the “Comments to the Author” section, enter your conflict of interest statement in the “Confidential to Editor” section, and submit your "Accept" recommendation.

Reviewer #1: All comments have been addressed

Reviewer #2: All comments have been addressed

**********

2. Does this manuscript meet PLOS Digital Health’s publication criteria? Is the manuscript technically sound, and do the data support the conclusions? The manuscript must describe methodologically and ethically rigorous research with conclusions that are appropriately drawn based on the data presented.

Reviewer #1: Yes

Reviewer #2: Yes

**********

3. Has the statistical analysis been performed appropriately and rigorously?

Reviewer #1: Yes

Reviewer #2: Yes

**********

4. Have the authors made all data underlying the findings in their manuscript fully available (please refer to the Data Availability Statement at the start of the manuscript PDF file)?

The PLOS Data policy requires authors to make all data underlying the findings described in their manuscript fully available without restriction, with rare exception. The data should be provided as part of the manuscript or its supporting information, or deposited to a public repository. For example, in addition to summary statistics, the data points behind means, medians and variance measures should be available. If there are restrictions on publicly sharing data—e.g. participant privacy or use of data from a third party—those must be specified.

Reviewer #1: Yes

Reviewer #2: No

**********

5. Is the manuscript presented in an intelligible fashion and written in standard English?

PLOS Digital Health does not copyedit accepted manuscripts, so the language in submitted articles must be clear, correct, and unambiguous. Any typographical or grammatical errors should be corrected at revision, so please note any specific errors here.

Reviewer #1: Yes

Reviewer #2: Yes

**********

6. Review Comments to the Author

Please use the space provided to explain your answers to the questions above. You may also include additional comments for the author, including concerns about dual publication, research ethics, or publication ethics. (Please upload your review as an attachment if it exceeds 20,000 characters)

Reviewer #1: By adding the Caesarian group this study has become meaningful science.

Reviewer #2: All my questions have been addressed.

**********

7. PLOS authors have the option to publish the peer review history of their article (what does this mean?). If published, this will include your full peer review and any attached files.

**Do you want your identity to be public for this peer review?**  If you choose “no”, your identity will remain anonymous but your review may still be made public.

For information about this choice, including consent withdrawal, please see our Privacy Policy.

Reviewer #1: No

Reviewer #2: No

**********
